# Supplementary material for: Healthy and Unhealthy Plant-Based Diets and Glioma in the Chinese Population
Source: Brain Sci. 2023 Sep 30;13(10):1401. doi: 10.3390/brainsci13101401 (PMC10605677; doi:10.3390/brainsci13101401)
Supplement: Supplementary file 1 [file brainsci-13-01401-s001.zip › Supplementary Material_Revision.pdf]

Supplementary Material

Table S1. The results of subgroup analysis.

Table S1. The results of subgroup analysis

Group[a]	Model 1[b]	*P*	Model 2[c]	*P*
**Age**				
**≤40(n=500)**				
PDI	1.02(0.99-1.05)	0.142	1.00(0.96-1.04)	0.995
hPD	1.01(0.98-1.04)	0.497	1.01(0.97-1.06)	0.488
uPD	1.06(1.03-1.08)	<0.001	1.09(1.06-1.13)	<0.001
>41(n=512)				
PDI	0.92(0.89-0.95)	<0.001	0.89(0.85-0.93)	<0.001
hPD	0.96(0.92-0.99)	0.011	0.96(0.92-1.00)	0.057
uPD	1.10(1.07-1.13)	<0.001	1.12(1.08-1.17)	<0.001
**Sex**				
**Male(n=568)**				
PDI	0.98(0.96-1.00)	0.075	0.93(0.90-0.97)	<0.001
hPD	0.97(0.94-1.00)	0.062	0.97(0.94-1.01)	0.140
uPD	1.06(1.03-1.08)	<0.001	1.10(1.07-1.13)	<0.001
**Female(n=444)**				
PDI	0.96(0.94-0.99)	0.015	0.95(0.90-0.99)	0.019
hPD	1.02(0.98-1.05)	0.350	0.99(0.94-1.04)	0.615
uPD	1.11(1.08-1.15)	<0.001	1.14(1.09-1.20)	<0.001
**BMI**				
**≤23.31(n=506)**				
PDI	0.98(0.96-1.01)	0.212	0.97(0.93-1.01)	0.095
hPD	1.02(0.99-1.05)	0.266	1.00(0.96-1.05)	0.887
uPD	1.07(1.04-1.09)	<0.001	1.10(1.06-1.14)	<0.001
**>23.31(n=506)**				
PDI	0.95(0.93-0.98)	0.001	0.92(0.88-0.95)	<0.001
hPD	0.96(0.93-0.99)	0.020	0.98(0.94-1.02)	0.230
uPD	1.09(1.06-1.12)	<0.001	1.11(1.07-1.15)	<0.001
**Education level**				
**Middle school and below(n=385)**				
PDI	0.94(0.91-0.98)	0.001	0.93(0.88-0.97)	0.002
hPD	0.98(0.94-1.02)	0.293	1.01(0.96-1.07)	0.673
uPD	1.12(1.08-1.15)	<0.001	1.15(1.09-1.20)	<0.001
**University and above(n=627)**				
PDI	0.98(0.96-1.01)	0.123	0.95(0.92-0.98)	0.001
hPD	0.99(0.96-1.01)	0.316	0.98(0.95-1.02)	0.329
uPD	1.05(1.03-1.08)	<0.001	1.08(1.05-1.12)	<0.001
**Household income**				

**<3,000 ¥/month(n=141)**				
PDI	1.00(0.95-1.06)	0.939	0.98(0.90-1.06)	0.583
hPD	1.01(0.95-1.07)	0.804	1.05(0.95-1.15)	0.375
uPD	1.05(0.99-1.10)	0.053	1.15(1.05-1.26)	0.003
**≥3,000 ¥/month(n=871)**				
PDI	0.97(0.95-0.99)	0.001	0.94(0.91-0.97)	<0.001
hPD	0.99(0.96-1.01)	0.293	0.98(0.96-1.01)	0.285
uPD	1.09(1.06-1.11)	<0.001	1.10(1.08-1.13)	<0.001
**Smoking status**				
**Never smoking(n=735)**				
PDI	0.98(0.96-1.00)	0.045	0.96(0.93-0.99)	0.009
hPD	0.99(0.97-1.03)	0.916	0.98(0.95-1.02)	0.285
uPD	1.08(1.05-1.10)	<0.001	1.10(1.07-1.13)	<0.001
**Smoking(n=277)**				
PDI	0.96(0.93-0.99)	0.021	0.90(0.86-0.95)	<0.001
hPD	0.97(0.93-1.01)	0.177	1.01(0.95-1.07)	0.797
uPD	1.07(1.04-1.11)	<0.001	1.13(1.08-1.18)	<0.001
**History of allergies**				
**Yes(n=113)**				
PDI	0.98(0.92-1.03)	0.405	0.92(0.84-1.01)	0.065
hPD	0.94(0.88-1.00)	0.062	0.95(0.87-1.03)	0.213
uPD	1.04(0.99-1.10)	0.136	1.07(1.00-1.15)	0.040
**No(n=899)**				
PDI	0.97(0.95-0.99)	0.004	0.95(0.92-0.97)	<0.001
hPD	0.99(0.97-1.02)	0.713	0.99(0.96-1.02)	0.651
uPD	1.08(1.06-1.10)	<0.001	1.11(1.08-1.14)	<0.001
**Family history of cancer**				
**Yes(n=259)**				
PDI	0.97(0.94-1.02)	0.214	0.94(0.89-1.00)	0.049
hPD	1.01(0.96-1.06)	0.732	0.98(0.92-1.04)	0.464
uPD	1.08(1.04-1.13)	<0.001	1.10(1.05-1.16)	<0.001
**No(n=753)**				
PDI	0.97(0.95-0.99)	0.011	0.95(0.92-0.98)	<0.001
hPD	0.99(0.96-1.01)	0.280	0.99(0.96-1.03)	0.743
uPD	1.07(1.05-1.10)	<0.001	1.11(1.08-1.14)	<0.001

a. Unconditional logistic regression model was used for sensitivity analysis.

b. Model 1: Unadjusted model through logistic regression.

c. Model 2: Adjusted for age, BMI, occupation, education level, household income, high-risk residential areas, smoking status, history of allergies, history of head trauma, family history of cancer, physical activity, and energy intake (except for corresponding hierarchical variables).
